# Methods and processes for development of a CONSORT extension for reporting pilot randomized controlled trials

**DOI:** 10.1186/s40814-016-0065-z

**Published:** 2016-05-20

**Authors:** Lehana Thabane, Sally Hopewell, Gillian A. Lancaster, Christine M. Bond, Claire L. Coleman, Michael J. Campbell, Sandra M. Eldridge

**Affiliations:** 1 Clinical Epidemiology and Biostatistics, McMaster University, Hamilton, Ontario Canada; 2 Oxford Clinical Trials Research Unit, Nuffield Department of Orthopaedics, Rheumatology and Musculoskeletal Sciences, University of Oxford, Oxford, UK; 3 Centre for Primary Care and Public Health, Queen Mary University of London, London, UK; 4 School of Health and Related Research, University of Sheffield, Sheffield, South Yorkshire UK; 5 Department of Mathematics and Statistics, Lancaster University, Lancaster, Lancashire UK; 6 Centre of Academic Primary Care, University of Aberdeen, Aberdeen, Scotland, UK

**Keywords:** CONSORT extension, Pilot randomized controlled trial, Reporting guideline

## Abstract

**Background:**

Feasibility and pilot studies are essential components of planning or preparing for a larger randomized controlled trial (RCT). They are intended to provide useful information about the feasibility of the main RCT—with the goal of reducing uncertainty and thereby increasing the chance of successfully conducting the main RCT. However, research has shown that there are serious inadequacies in the reporting of pilot and feasibility studies. Reasons for this include a lack of explicit publication policies for pilot and feasibility studies in many journals, unclear definitions of what constitutes a pilot or feasibility RCT/study, and a lack of clarity in the objectives and methodological focus. All these suggest that there is an urgent need for new guidelines for reporting pilot and feasibility studies.

**Objectives:**

The aim of this paper is to describe the methods and processes in our development of an extension to the Consolidated Standards of Reporting Trials (CONSORT) Statement for reporting pilot and feasibility RCTs, that are executed in preparation for a future, more definitive RCT.

**Methods/design:**

There were five overlapping parts to the project: (i) *the project launch*—which involved establishing a working group and conducting a review of the literature; (ii) *stakeholder engagement*—which entailed consultation with the CONSORT group, journal editors and publishers, the clinical trials community, and funders; (iii) a *Delphi process*—used to assess the agreement of experts on initial definitions and to generate a reporting checklist for pilot RCTs, based on the 2010 CONSORT statement extension applicable to reporting pilot studies; (iv) a *consensus meeting*—to discuss, add, remove, or modify checklist items, with input from experts in the field; and (v) *write-up and implementation—*which included a guideline document which gives an explanation and elaboration (E&E) and which will provide advice for each item, together with examples of good reporting practice. This final part also included a plan for dissemination and publication of the guideline.

**Conclusions:**

We anticipate that implementation of our guideline will improve the reporting completeness, transparency, and quality of pilot RCTs, and hence benefit several constituencies, including authors of journal manuscripts, funding agencies, educators, researchers, and end-users.

## Background

Feasibility and pilot studies are essential components of planning or preparing for a larger randomized controlled trial (RCT). They are intended to provide useful information about the feasibility of running the main RCT [1, 2] with the goal of reducing uncertainty and thereby increasing the chance of successfully conducting the main RCT. They are also useful preliminary studies that other researchers can learn from when developing their own study designs to enhance their approach or avoid similar pitfalls. However, many journals do not have a specific publication policy for these types of study or consider them a priority, and it has been shown that there are serious inadequacies in how pilot and feasibility studies are reported [1–6]. First, the dearth of published research describing pilot/feasibility studies suggests that only a minority of them actually reach publication; additionally, when they are published, a wide variety of terms are used to describe them. Second, only a small percentage of this minority of published pilot and feasibility studies explicitly state that they are intended as preparation for a larger RCT. Third, in many instances, the objectives of pilot and feasibility studies are unclear or are mis-specified as being the same as in the main RCT. Lastly, methodological features of pilot and feasibility studies are often inappropriately reported in the same format as in the main RCT. Reasons for these deficiencies include not only the lack of explicit publication policies for pilot and feasibility studies in many journals [3, 4] but also unclear definitions of what actually constitutes a pilot or feasibility RCT/study [2, 6] and confusion about what the objectives and methodological focus ought to be in such studies [1–3]. All these suggest that there is an urgent need for the development of new guidelines for reporting of pilot and feasibility studies. Given the importance of these studies in preparing for future definitive trials, we anticipate that the guideline will help to address the prevailing flaws in their conduct and reporting, leading to superior-quality pilot trials and enhanced feasibility for the main RCTs.

Initial discussions on developing reporting guidelines for feasibility and pilot studies occurred at the annual scientific meeting of the Society for Academic Primary Care (SAPC) held in Bristol (UK) on June 6–8, 2011, during a workshop on “*Pilot and feasibility studies: How best to obtain pre-trial information and publish it*”, organized and led by Sandra Eldridge, Gillian Lancaster, and Sally Kerry and attended by Christine Bond. The workshop was intended to clarify the aims of pilot and feasibility studies, improve understanding of the particular requirements of these studies (including specification of their key objectives), and discuss how to report them appropriately. It was proposed (after the workshop) that reporting guidelines for feasibility and pilot studies would be helpful as a template for researchers, reviewers, and editors to use when preparing or reviewing papers for publication; they would also provide guidance to funders and policy-makers who review grant or funding proposals for feasibility and pilot studies.

Arising from this workshop, SE, GL, and CB engaged other collaborators in the area (MJC, LT, SH), and the group embarked together on a programme of research focusing on the reporting of feasibility and pilot studies. This paper reports part of that work: the methods and processes used in the development of a consolidated standards of reporting trials (CONSORT) extension guideline for reporting randomized pilot and feasibility studies. The guideline focuses on reporting of pilot and feasibility RCTs, using the 2010 CONSORT Statement [7] as the starting point. The original CONSORT guideline aimed to improve the reporting of two-arm parallel group RCTs [8] and was later extended to cover other types of designs: cluster randomized trials [9], non-inferiority and equivalence trials [10], pragmatic trials [11], and N-of-1 trials [12]. A variety of clinical areas has also been discussed, including the following interventions: herbal medicinal [13], non-pharmacological [14], and acupuncture [15]. Finally, related types of data have been described, including the following: patient-reported outcomes [16], harms [17], abstracts [18], and RCT protocols [19].

### Aims

The aim of this paper is to describe the methods and processes for development of our CONSORT extension for reporting feasibility and pilot RCTs that are executed in preparation for a future definitive large-scale RCT. Reporting guidance for other types of pilot and feasibility studies—which include non-randomized and qualitative pilot and feasibility studies—will be a focus of future work.

## Methods and processes

**Fig. 1:**
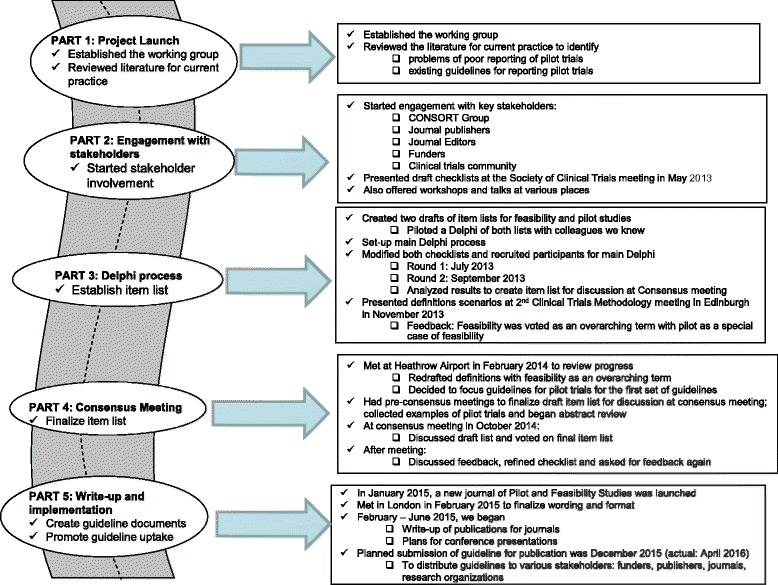
Development of the CONSORT extension to pilot trials guideline

We followed previously recommended methods and processes for developing, disseminating, and implementing consensus reporting guidelines [20–22]. Briefly, these included a series of activities: (1) *project launch*—which included establishing the working group, identifying the need for the guideline, performing a literature review of current practice, and drafting an initial list of items and starting to seek funding support for the project; (2) engagement with stakeholders—which included identifying potential participants for a Delphi study and face-to-face consensus meeting and early presentations of potential items to gain feedback at conferences and workshops; (3) conducting the Delphi study, including a pilot Delphi and set-up of the questions online and presentation of the results at a methodology meeting of trialists; (4) *a consensus meeting*, to present the results of the literature review and the Delphi study and to discuss the revised list of checklist items; (5) write-up and implementation including creating the guideline, addressing feedback from users, establishing the explanation and elaboration (E&E) document, and devising a publication strategy; and (6) *post-publication activities*—which covers encouraging guideline uptake and endorsement, updating the guideline, and evaluating impact. These methods or their variations and adaptations have been used in development of other similar guidelines [7, 9–20]. Figure 1 illustrates the five parts of the development process for this guideline: (i) the project launch, (ii) stakeholder engagement, (iii) a modified Delphi process, (iv) a consensus meeting, and (v) write-up and implementation. It does not yet include post-publication activities where we will engage in dissemination and endorsement, as we have not yet reached this stage. The development process is described below. But it should be noted, however, that the process was iterative—repeating some part(s) as necessary—rather than linear.

### Part 1: project launch

#### Establishing a working group

After the SAPC meeting in June 2011, our (SE, GL, CB) first step was to establish a working group to lead the process of developing the reporting guidelines. The core makeup of the group included people with experience in conducting and publishing methodological work on pilot and feasibility RCTs (CB, SE, GL, LT, MC) and methodologists and statisticians with expertise in the design and reporting of RCTs and in reviewing funding or ethics applications (SE, GL, LT, MC). A member of the CONSORT group (SH) was invited and agreed to join the group during the Delphi process. The group communicated regularly throughout the process via a number of face-to-face and virtual meetings (by teleconference or Skype) and email discussions. CC joined the group when she was appointed to a National Institute of Heath Research (NIHR) research methods fellowship focusing on pilot studies supervised by SE in 2013.

#### Review of the literature

We first reviewed the literature to assess the quality of pilot and feasibility studies that had been published in major medical journals (*Lancet*, *the BMJ*, *the New England Journal of Medicine*, *JAMA*). These are amongst the leading medical journals that have been included in previous systematic reviews or surveys of the reporting of pilot studies [1, 3]. We included articles identified in the searches by Lancaster et al. [1] and Arain et al. [3], as well as examples used in previous workshops conducted by some of the working group members. We assessed whether clear statements had been made with respect to the following: whether the article concerned a pilot or feasibility study; feasibility objectives; and whether they stated that the feasibility or pilot study was in preparation for a larger RCT. We also reviewed existing definitions of and reporting guidelines for pilot and feasibility studies [1, 2, 23].

### Part 2: stakeholder engagement

We engaged several groups of stakeholders in the process:

- *The CONSORT group*: As noted earlier, the guideline was developed with involvement of the CONSORT group. As with other CONSORT-related guidelines, the inclusion of a CONSORT Group member (SH) was intended to ensure consistency in the use of recommended methods in the development, dissemination, and implementation of high quality reporting guidelines [24].
- *Clinical Trials Community*: Our first engagement of the clinical trials community was at the Annual Meeting of the Society for Clinical Trials in Boston in May 2012, where we presented early work from the Delphi study (see later). This was organized as an invited session at the meeting, and there were about 40 attendees. The presentation focused on problems in the reporting of pilot and feasibility studies and the need to develop guidance to improve the situation [25].

A second opportunity to engage a larger clinical trials community was at the 2nd Clinical Trials Methodology Conference in November 2013 in Edinburgh, Scotland. This was an open session, and the discussion focused mainly on the definitions of pilot and feasibility studies (see “Part 3: the Delphi process” section later).

Over the course of the project, we have also delivered a number of workshops and talks on feasibility and pilot studies and sought feedback from the research community. Overall, the reactions have supported the idea of developing a CONSORT-type reporting guidelines for feasibility and pilot studies. We recognize that there are differences of opinion about the definitions of these studies, particularly as they reflect various user groups and theoretical perspectives, as we report elsewhere [26].

- *Journal Editors and Publishers*: We engaged editors of prominent journals known to published pilot and feasibility studies including the *BMJ Open*, *Journal of Clinical Epidemiology*, *Clinical Trials*, and *BMC Trials*. The selection of the journal editors was pragmatic: (i) our knowledge of publication of pilot and feasibility studies led us to believe that these journal editors would be interested in the work; (ii) the working group members were already serving on the editorial boards of some of these journals and had sent out personal invitations to the editors; and (iii) these editors were available to attend the consensus meeting. We also engaged several publishers including BioMed Central and the *BMJ*. We will continue to engage other journal editors and publishers to ensure wider awareness and endorsement of our guideline upon their completion. It has been shown that formal endorsement of a guideline by journals is a strong determinant of its adoption and subsequent adherence to it [27].
- *Funders*: We approached several funding agencies, including the Medical Research Council UK, the Canadian Health Research Institutes, and the Chief Scientist Office, Scottish Government, to engage them in providing financial support for the development of the guideline. These are amongst the major agencies that have financially supported pilot studies through the national or international funding competitions. Our own project was subsequently funded in part by the Queen Mary University of London, the University of Sheffield, the Chief Scientist Office in Scotland, the NIHR Research Design Services London and South East and the NIHR Statisticians Network.

The project was registered on the Enhancing the QUAlity and Transparency Of health Research (EQUATOR) Network website [28].

### Part 3: the Delphi process

The Delphi process is iterative and provides a structured collection of input, information, and feedback from participants by using a series of survey questionnaires. Typically, each questionnaire is refined based on comments from previous iterations [29]. Generally considered to be one of the central features in developing reporting guidelines [21, 22], the Delphi method has been widely used in this way [7, 9–20]. The objectives of our Delphi process were (a) to evaluate the agreement of the various participants (approximately 100 stakeholders in total, including trialists, methodologists, and statisticians) with respect to our initial definitions of feasibility and pilot studies; (b) to assess their agreement on two reporting checklists, one for pilot studies and the other for feasibility studies (see rationale below), with the initial items being based on the current (2010) CONSORT Statement [7]; (c) to elicit any further items or changes to items that the participants thought might be important; and (d) to identify which items the Delphi participants felt were the most important. We received research ethics approval for the Delphi study from the University of Sheffield Research Ethics Committee.

Personal networks allowed us to identify individual participants who were involved in, or interested in, pilot and feasibility studies. We also sent invitations to people on contact lists of the following: Canadian Institutes of Health Research, the Biostatistics Section of the Statistical Society of Canada, the American Statistical Society, UK NIHR, Medical Research Council (MRC) Methodology Hubs, the International Society for Clinical Biostatistics, the UK Clinical Trials Units Network, the Society for Clinical Trials, and the World Association of Medical Editors.

Based on the literature review described earlier, the current CONSORT Statement [7], and the mutually exclusive definitions of pilot and feasibility studies articulated by the UK National Institute of Health Research, we created two versions of the checklist—one for feasibility studies and the second for pilot studies (Table 1).

**Table 1:** Drafts of the initial proposed checklists for feasibility and pilot studies

Item no. (original CONSORT)	Feasibility studies	Pilot studies
Abstract		
1a	Identification as feasibility study in title	Identification as pilot in the title; randomized in title if used
1b	Summary of study design, methods, results, and conclusions	Summary of study design, methods, results, and conclusions
Background		
2a	Scientific background and explanation of rationale for feasibility study	Scientific background and explanation of rationale for pilot study
2b	Key aims and objectives of feasibility study	Key aims and objectives of pilot study
2c	Description of type of trial planning for (e.g. drug development, health services, community intervention, cluster trial, etc.)	Description of type of trial planning for (e.g. drug development, health services, community intervention, cluster trial, etc.)
Methods		
3a1	Description of design of feasibility study covering all objectives (may have several components addressing different objectives which all need to be described); adequate descriptions of how each objective is to be addressed and any relevant thresholds for successful implementation of component(s)	Description of design of pilot study (should differ from that of main study because the aim is not to test effectiveness—thus, data collected and types of analysis may differ); how differs from main study; thresholds for success/proceeding to main trial
3a2	Description of criteria used to judge feasibility (often a threshold, for example for recruitment rates)	Description of criteria used to judge whether to proceed with main trial (often a threshold, for example for recruitment rates)
3b	Important changes to methods, outcomes, eligibility criteria, etc. during the study, with reasons (changes are sometimes made during feasibility studies because lack of feasibility is identified early on in a study)	Important changes to methods, outcomes, eligibility criteria, etc. during the study, with reasons (changes are sometimes made during pilot studies because a difficulty with a particular aspect is identified fairly early on)
4a	Specify how participants were selected for each component, how many refused, if volunteers; eligibility criteria if any	Eligibility criteria for participants, how many refused; if cluster trial pilot address issues around bias and contamination
4b	Settings and locations where the data were collected	Settings and locations where the data were collected
	Description of how potential biases in the main trial are being explored in the feasibility study (potential biases: selection, detection, performance, attrition)	Description of how potential biases in the main trial are being explored in the pilot study (potential biases: selection, detection, performance, attrition)
5a	Detailed description of intervention to be tested in main trial, specifying assessor, administration, duration, quality control (e.g. calibration, training), etc. to be used in any feasibility assessments	Detailed description of intervention being assessed in pilot study, including assessor(s), control group if using, administration, duration, quality control (e.g. calibration, training), etc. as appropriate
6a	Specify assessments or measurements to be made to address each objective, including how and when they were made (each component of the study should be addressed); include how feasibility of descriptive components will be addressed	Specify range of measurements to be taken including main outcome measure, secondary outcome measures, if can be identified, recruitment and consent rates, etc.
6b	Details of qualitative analysis if appropriate, cost-effectiveness	Details of pre-trial modelling criteria
7a	Appropriate justification for sample size for each component (unlikely to include a sample size calculation, more likely to be a pragmatic decision)	Appropriate justification for sample size (does not need to include a formal trial sample size calculation but may include other types of sample size calculations)
8a	Details of administration of or qualitative work related to randomisation	Method used to generate random allocation sequence (if randomized pilot study); description of how it will be administered; details of any restrictions; who generated the random allocation sequence, who enrolled participants, and who assigned participants to interventions
11a	If done, details of how blinding was considered and used	If done, who was blinded, e.g. after assignment to interventions (for example, participants, care providers, those assessing outcomes) and how
11b	If relevant, description of how the interventions are to be made similar and details of any testing done	If relevant, description of the similarity of interventions
12a	Any statistical methods used in the analysis of each component (if relevant)	Statistical methods used to summarize and compare groups for primary and secondary outcomes; estimates of effect size with confidence intervals; no hypothesis testing is recommended
12b	Methods for additional analyses not addressing key objectives, such as adjusted analyses and cost-effectiveness with justification	Methods for additional analyses not addressing key objectives, such as adjusted analyses and cost-effectiveness with justification
Results		
13a	Description of participants and numbers for components being assessed with flow diagram if appropriate	Description of participants and numbers for components being assessed with flow diagram if appropriate; if randomisation used for each group, the numbers of participants who were randomly assigned, received intended treatment, and were assessed for the outcome(s)
13b	Losses and exclusions for each component being tested, including reasons	Losses and exclusions including reasons; if randomisation used for each group
14a	Dates defining the periods of recruitment and follow-up for each component	Dates defining the periods of recruitment and follow-up
14b	Why recruitment to the study ended or was stopped prior to the planned end of study (if relevant)	Why recruitment to the study ended or was stopped prior to the planned end of study (if relevant)
15	Summary of people or samples used for each component tested	A table showing baseline demographic and clinical characteristics for participants in the study; if randomisation used for each group
16	For each component, number of participants (denominator) or samples included in each analysis or data summary	For study participants and each group if randomized, number of participants (denominator) included in each analysis or data summary; if randomized whether the analysis was by original assigned groups
17a	For all components tested ensure results match objectives; if relevant estimated effect size and its precision (such as 95 % confidence interval); for binary outcomes, presentation of both absolute and relative effect sizes is recommended	For all parameters and outcomes tested ensure results match objectives; estimated effect size and its precision (such as 95 % confidence interval); for binary outcomes, presentation of both absolute and relative effect sizes is recommended
18	Results of any other analyses performed, including adjusted analyses, distinguishing pre-specified from exploratory	Results of any other analyses performed, including subgroup analyses and adjusted analyses, distinguishing pre-specified from exploratory
19	All important harms or unintended effects; detail and discussion; patient questionnaires used to assess safety, adverse events, harms, etc.	All important harms or unintended effects; detail and discussion; patient questionnaires used to assess safety, adverse events, harms, etc.
Discussion		
20	Limitations addressing sources of potential bias, changes to components, imprecision of estimates, multiplicity of analyses, etc.	Limitations addressing sources of potential bias, changes to components, imprecision of estimates, multiplicity of analyses etc. and changes to pilot study protocol
21	Generalisability of the findings to other studies; transferable information (external validity, applicability), etc.	Generalisability of pilot work to other studies; is larger trial needed; transferable information (external validity, applicability), etc.
22a	Interpretation consistent with results for each component tested, balancing benefits and harms, and considering other relevant evidence	Interpretation consistent with results for pilot study, balancing benefits and harms, and considering other relevant evidence; will main trial go ahead; how it will be designed
22b	Assessment of feasibility of each component	Changes to main study protocol
23	Not applicable	Registration number and name of trial registry
24	Research review committee approval, ethics approval and consent to participate	Research review committee approval, ethics approval and consent to participate; where the trial protocol can be accessed, if available
25	Sources of funding and other support	Sources of funding and other support

The Delphi survey comprised three sections: participant demography, feedback on the NIHR and MRC definitions of feasibility and pilot studies [23, 26], and the two checklist items (Table 1). The survey was produced in an online format using CLINVIVO software and distributed through a weblink sent in an email.

The Delphi process was carried out between June and October 2013. It was done in two phases. *Phase 1* was the pilot phase. We piloted the Delphi survey using a “think-aloud” approach on 13 colleagues working at our own institutions. The purpose of this phase was to evaluate the questionnaire for face and content validity, usability, and clarity of its items.

**Fig. 2:**
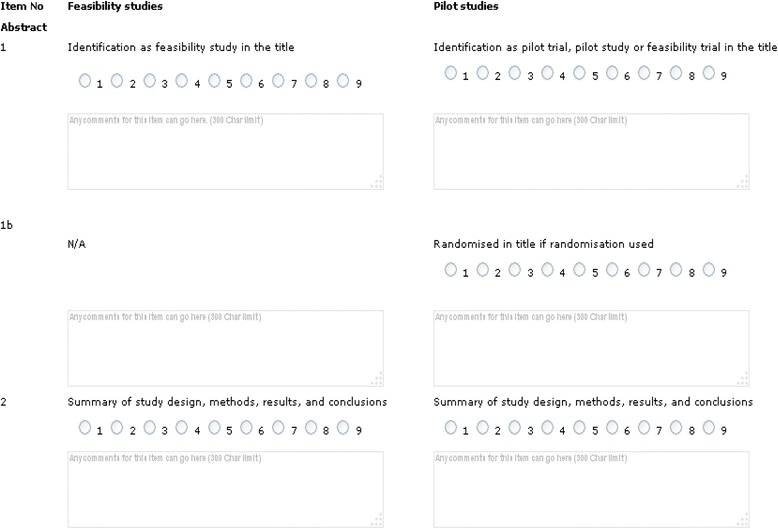
An example of Delphi presentation for the two checklists
  Overall, 93/100 participants responded to the online survey. Table 2 provides a summary of the demographic characteristics of the respondents.

*Phase* 2 was the main Delphi study, which was conducted in two rounds using an online survey conducted by Clinvivo.

i. *First round*: We invited participants to declare an interest in the study to Clinvivo by emailing fapsdelphi@clinvivo.com. Clinvivo then replied by sending a personalized link to the round. The round opened on July 26, 2013 and closed on August 27, 2013. Invitations were sent to all those who had expressed an interest up to and including August 26.
  The two checklists are presented in Table 1. Most items were identical or similar in the two lists. The participants rated items on a nine-point scale, ranging from 1 = “not at all appropriate” to 9 = “completely appropriate”. Participants could also write comments under each rating (Fig. 2).

**Table 2:** The demographic characteristics of the main Delphi study: *n* = 93

Demographic variables	Levels	Statistics: *n* (%)
Primary discipline/job title	Statistician	53 (56.99 %)
	Epidemiologist	2 (2.15 %)
	Health service researcher	13 (13.98 %)
	Clinician scientist	4 (4.30 %)
	Clinical trial methodologist	5 (5.38 %)
	Others	16 (17.20 %)
Country/region of residence	UK	38 (40.86 %)
	Europe	31 (33.33 %)
	North America	23 (24.73 %)
	Others^a^	1 (1.08 %)
Key role in clinical trials^b^	Statistician	56 (60.22 %)
	Methodologist	12 (12.90 %)
	Principal/chief investigator	27 (29.03 %)
	Co-investigator	12 (12.90 %)
	Trial manager	7 (7.53 %)
	Research assistant	14 (15.05 %)
Experience in trials (years)	0–5	16 (17.20 %)
	6–10	25 (26.88 %)
	11–15	13 (13.98 %)
	16–20	18 (19.35 %)
	>20	21 (22.58 %)
Ever been involved in write-up of study in preparation for an RCT	Yes	61 (65.59 %)
	No	32 (34.41 %)

^a^Japan

^b^Levels are not mutually exclusive

*RCT* randomized controlled trial
ii.) *2nd round*: Participants who had completed the first round were sent an email link to the second round on September 24. Reminders to complete was sent on October 7 and 14. The round closed on midnight, at the International Date Line, on October 15.
  In this round, participants were asked to review tables of the scores of histograms of pilot and feasibility items for which 70 % or more of the panel had rated using the two highest appropriateness scores (i.e. 8 and 9). They could then make additional comments on these items. Participants were also asked to review the remaining items that had been rated slightly lower in terms of appropriateness, and for each item, they were asked to indicate whether they thought the item should be kept, discarded, or whether they were unsure or had no opinion.
  Participants were also asked whether they thought any reporting aspects had been missed and should be included in a checklist, and separately whether a pilot study checklist would be suitable for phase I studies, phase II studies, internal pilots, and external pilots; and whether a feasibility checklist would be suitable in the context of qualitative work, and what they would regard as quantitative work. In each case, participants could rate items as suitable, unsure/no opinion, or not suitable. Finally, participants could add comments on any other aspects about the suitability of pilot and feasibility study checklists. About 85 % (79/93) of the respondents participated in the second round.

Overall, the Delphi results showed a strong agreement on checklist items both for pilot and feasibility studies. However, there was substantial disagreement about the definitions of pilot and feasibility studies and the distinction between them.

At the Edinburgh meeting, we presented four *propositions regarding definitions* and preliminary Delphi results [26]. The main outcome from the discussions was that participants unanimously suggested that we should begin by developing only one reporting checklist. At this stage, it was unclear how wide the scope of this checklist would be, though a strong steer from the meeting was not to make it too wide.

### Part 4: the consensus meeting

*Prior to the consensus meeting*: In February 2014, our group had a face-to-face meeting in London, UK, to review progress. Based on the feedback from all the stakeholder engagements and Delphi process results, we redrafted our definitions, with feasibility as an overarching term, and we agreed to focus on reporting guidelines for pilot/feasibility RCTs as our next step. We finalized the draft list of items for a reporting guideline to be discussed at the consensus meeting. At this stage, we also confirmed with the CONSORT group that our checklist would be included as an official CONSORT checklist extension.

*Consensus meeting*: We held a 2-day meeting in Oxford, UK, on October 27–28, 2014, to seek feedback on the proposed items to be included in the guideline, and its scope. We invited a group of international stakeholders (*n* = 26) representing different professional sectors (academic, pharmaceutical, journal editors, publishers, funding bodies) and different clinical RCT roles (such as trialists, methodologists, statisticians, and clinicians).

Using approaches that were similar to those used in previous consensus meetings for other guidelines [7, 9–20], participants were presented in advance of the meeting with the results of the literature review and the Delphi survey. Working group members presented the background and an update on work done to date, in order to facilitate the discussions. We also presented a penultimate version of the checklist—based on the Delphi process and feedback from the earlier stakeholder engagement meetings. The meeting was audiotaped, and formal minutes were subsequently prepared and circulated to all attendees.

The key recommendations that emerged were as follows:

- Modify items: It was recommended that 24 items should be modified. These modifications were primarily to prefix all references to “trial” with “pilot” to clearly indicate that the information being reported is about the pilot RCT, and not the main RCT. As in previous CONSORT extensions [9–19], some of the recommended changes begin with “if relevant” or “when applicable”, to show that some information which authors are being asked to report might not be relevant or applicable for their particular pilot RCT.
- Add new items: Four new items were suggested as follows. *Participants*: how participants should be identified and consented; *outcomes*: if applicable, criteria used to judge whether, or how, to proceed with a future definitive RCT; *limitations*: implications for progression from the pilot to a future definitive RCT, including any proposed amendments or modifications to the protocol of the main RCT; and *other information*: if ethical approval/research review committee approval was confirmed, with a reference number.
- Remove items: It was recommended that, beyond an item our group had already suggested, removing one further item should be removed, *Methods for additional analyses*, *such as subgroup analyses and adjusted analyses.* Participants felt strongly that this item was not applicable to pilot RCTs, because such analyses would be about hypothesis testing or generation—which is not the focus of a pilot RCT.

### Part 5: write-up, dissemination, and implementation

Following the consensus meeting, we continued to refine the content and wording of the items by virtual group discussion and by involving those who had attended the meeting, to ensure they reflected the decisions that had been made. There was second working group meeting in London, UK, on January 12, 2015. We discussed the feedback from the consensus meeting in detail and outlined strategies to complete the write-up of the guideline, including plans for dissemination and implementation in order to maximize its adoption by various journals, professional associations and the clinical trial community.

As with previous guidelines [7, 9–20], our guideline statement will be published with a detailed Explanation and Elaboration (E&E) document that will provide an in-depth explanation of the scientific rationale for each recommendation, together with an example of clear reporting for each item. We have sought feedback from the consensus conference participants on the E&E document to ensure that it accurately reflects the discussions and decisions that were made during the meeting. To widely disseminate the guideline, we will publish in peer-reviewed journals and do presentations and workshops at conferences and other venues. We also plan to seek endorsement of the guideline by journal editors. Research has shown that formal endorsement and adoption of the CONSORT Statement by journals is associated with improved quality of reporting [26]

## Discussion

This article has described the methods and processes that we have used to develop a CONSORT extension for reporting of pilot/feasibility RCTs—using the 2010 version of the CONSORT Statement as its basis [7]. The work actually began with a broader mandate, to develop guidelines for feasibility and pilot studies. However, after receiving feedback from the research community, we have started with a narrower focus of firstly developing a set of guidelines for reporting feasibility and pilot RCTs. We have attempted to use the best available and evidence-based methods [21, 22], similar to those used by other guideline developers [7, 9–20]. These include establishing a working group to lead the project; conducting a systematic review of the literature to determine current practice and identify available guidelines; applying an online Delphi survey on the initial list of items to be included in the guideline; holding a consensus meeting attended by various stakeholders to finalize the list; and creating a dissemination plan to enhance uptake for the guideline. In addition, because of the perceived differences of opinion about the definitions of feasibility and pilot studies, we found that an ongoing discussion amongst the research community over a considerable period was invaluable for validating the direction of our work.

In this paper, we have provided detailed descriptions of the methods and processes that we used to develop our guideline. These details are intended to provide readers with enough information to assess the quality and validity of the methods used to develop the CONSORT extension to pilot/feasibility RCTs guideline. We applied approaches, methods, and processes that had been used previously by guideline developers, to ensure that the foundation for and development of our own guideline was truly evidence-based. As with previous guidelines [7, 9–19], we involved a wide spectrum of stakeholders and participants representing different sectors, perspectives, areas of expertise, and experiences with trials—both in the Delphi process and the consensus meeting. The participants in the Delphi surveys included (bio)statisticians, clinicians, health services researchers, regulatory staff, primary care practitioners, to mention a few. A potential limitation is that the views of non-statisticians may not have been adequately represented since the majority of the participants were statisticians. The participants in the consensus meeting came from different stakeholder groups representing professional sectors (academic, pharma, journal editors, publishers, funding bodies) and different clinical RCT roles (such as trialists, methodologists, statisticians, and clinicians). Prior to the consensus meeting, we had several Skype and face-to-face discussions and presentations at several professional conferences, to gather data and feedback. These steps were preceded by an extensive review of the literature to assess the reporting of pilot and feasibility trials.

We also spent a considerable amount of time debating alternative definitions of feasibility and pilot trials, and we used the Delphi study along with discussions at conferences, and the expert consensus meeting, to get feedback on them. This led to the development of a framework, in which pilot studies are viewed as a subset of feasibility studies [26]. Within this framework, a feasibility study is defined as a study asking “*whether something can be done, should we proceed with it, and if so, how*” [26]. In contrast, “*a pilot study asks the same questions but also has a specific design feature: in a pilot study a future study, or part of a future study, is conducted on a smaller scale*” [26]. The framework and the resulting definitions became essential elements in the development process of the guideline.

We hope that our guideline will improve the reporting of pilot/feasibility RCTs. We have already liaised with editors of some key clinical journals, and we also plan to embark on a campaign to get more journals to endorse the guideline. The intent is to target several groups: authors of journal manuscripts, who can use it as an outline for reporting results of their pilot/feasibility RCTs; manuscript reviewers, who can use it as template to evaluate reports of pilot/feasibility RCTs; funding agencies, for use as a foundation to create funding programmes for and evaluation of pilot and feasibility RCT proposals; educators, for use as a tool for training students and researchers about the unique nature of pilot RCT methodology and reporting; and end-users, for use as a tool to identify relevant pilot RCTs—that provide evidence about feasibility to inform their planning of main RCTs or other pilot/feasibility RCTs.

Like all reporting guidelines, ours will require re-evaluation and revisions over time—to ensure that it is kept up to date with evolving research and knowledge on pilot and feasibility trials.

One major outcome of this work is the setting up of a new journal by BioMed Central, *Pilot and Feasibility Studies* (http://pilotfeasibilitystudies.biomedcentral.com/), which was launched on January 12, 2015. It provides a platform for publishing these types of studies and constitutes a much-needed place for researchers to share their work and ideas on all aspects of the design, conduct, and reporting of pilot and feasibility studies in health or biomedical research. While this is an important achievement on its own, we hope that our guideline will also be a catalyst for the establishment of better publication practices and editorial policies regarding the reporting of pilot and feasibility trials—a deficiency that has been noted previously [1, 3]. As of March 19, 2016, the new journal has received over 70,000 unique web accesses, published 61 papers, of which 34 are protocols, 21 report the results of pilot or feasibility studies/trials, and 6 are reviews, commentaries, or methods papers. These statistics suggest that investigators are indeed using the journal as an outlet for publishing their pilot or feasibility works.

### Participants at the Oxford 2014 consensus meeting

Doug Altman^1^, Colin Begg^2^, Frank Bretz^3^, Marion Campbell^4^, Erik Cobo^5^_,_ Peter Craig^6^, Peter Davidson^7^, Trish Groves^8^, Freedom Gumedze^9^, Jenny Hewison^10^, Allison Hirst^11^, Pat Hoddinott^12^, Sallie Lamb^13^, Tom Lang^14^, Elaine McColl^15^, Alicia O’Cathain^16^, Daniel R Shanahan^17^, Chris Sutton^18^, Peter Tugwell^19^,

### Working group

Christine Bond^20^, Michael Campbell^21^, Claire Coleman^22^, Sandra Eldridge^22^, Sally Hopewell^23^, Gillian Lancaster^24^, Lehana Thabane^25^

1. Centre for Statistics in Medicine, Nuffield Department of Orthopaedics, Rheumatology and Musculoskeletal Sciences, University of Oxford, Oxford, UK
2. Department of Epidemiology and Biostatistics, Memorial Sloan Kettering Cancer Center, New York, NY, USA
3. Statistical Methodology and Consulting, Novartis Pharma AG, Basel, Switzerland
4. Health Services Research Unit, University of Aberdeen, Polwarth Building, Foresterhill, Aberdeen, UK
5. Department of Statistics and Operations Research, UPC, Barcelona-Tech, Spain
6. MRC/CSO Social and Public Health Sciences Unit, University of Glasgow, Glasgow, UK
7. National Institute for Health Research, Evaluation, Trials, and Studies Coordinating Centre, University of Southampton, Southampton, UK
8. BMJ, BMA House, London, UK
9. Department of Statistical Sciences, University of Cape Town, Cape Town, South Africa
10. Leeds Institute of Health Sciences, University of Leeds, Leeds, UK
11. IDEAL Collaboration, Nuffield Department of Surgical Sciences, University of Oxford, Oxford, UK
12. Nursing Midwifery and Allied Health Professionals Research Unit, Faculty of Health Sciences and Sport, University of Stirling, Scotland, UK
13. Oxford Clinical Trials Research Unit, Nuffield Department of Orthopaedics, Rheumatology and Musculoskeletal Sciences, University of Oxford, Oxford, UK
14. Tom Lang Communications and Training International, Kirkland, Washington, USA
15. Newcastle Clinical Trials Unit and Institute of Health & Society, Newcastle University, Newcastle Upon Tyne, UK.
16. School of Health and Related Research, University of Sheffield, Sheffield, UK
17. BioMed Central, London, UK
18. School of Health, University of Central Lancashire, Preston, UK
19. Department of Medicine, University of Ottawa, Ottawa, Ontario, Canada
20. Centre of Academic Primary Care, University of Aberdeen, Aberdeen, Scotland, UK
21. School of Health and Related Research, University of Sheffield, Sheffield, South Yorkshire, UK
22. Centre for Primary Care and Public Health, Queen Mary University of London, London, UK
23. Oxford Clinical Trials Research Unit, Nuffield Department of Orthopaedics, Rheumatology and Musculoskeletal Sciences, University of Oxford, Oxford, UK
24. Department of Mathematics and Statistics, Lancaster University, Lancaster, Lancashire, UK
25. Clinical Epidemiology and Biostatistics, McMaster University, Hamilton, Ontario, Canada
