# Erratum to: Methods and processes for development of a CONSORT extension for reporting pilot randomized controlled trials

**DOI:** 10.1186/s40814-016-0078-7

**Published:** 2016-07-19

**Authors:** Lehana Thabane, Sally Hopewell, Gillian A. Lancaster, Christine M. Bond, Claire L. Coleman, Michael J. Campbell, Sandra M. Eldridge

**Affiliations:** 1 Clinical Epidemiology and Biostatistics, McMaster University, Hamilton, Ontario Canada; 2 Oxford Clinical Trials Research Unit, Nuffield Department of Orthopaedics, Rheumatology and Musculoskeletal Sciences, University of Oxford, Oxford, UK; 3 Centre for Primary Care and Public Health, Queen Mary University of London, London, UK; 4 School of Health and Related Research, University of Sheffield, Sheffield, South Yorkshire UK; 5 Department of Mathematics and Statistics, Lancaster University, Lancaster, Lancashire UK; 6 Centre of Academic Primary Care, University of Aberdeen, Aberdeen, Scotland, UK

## Erratum

After publication of the original article [1], it came to the authors’ attention that there was an error in the Discussion section. In the final paragraph, “70,000 submissions” should have read “70,000 unique web accesses”. The article has been updated to rectify this error.

## References

[1] Thabane L, Hopewell S, Lancaster GA, Bond CM, Coleman CL, Campbell MJ, Eldridge SM (2016). Methods and processes for development of a CONSORT extension for reporting pilot randomized controlled trials. Pilot and Feasibility Studies.

